# Not All Awe Is Equal: Divergent and Unstable Effects of Positive and Negative Awe on Aggressive Behavior

**DOI:** 10.3390/bs16050625

**Published:** 2026-04-22

**Authors:** Fen Ren, Wei Liu

**Affiliations:** 1 School of Education and Psychology, University of Jinan, No. 336, West Nanxinzhuang Road, Jinan 250022, China; 2 Inner Mongolia Student Bullying Prevention Research Center, Tongliao 028000, China; 3 The School of Education, Guangzhou University, Guangzhou 510006, China

**Keywords:** awe, proactive aggression, empathy, bug-killing paradigm

## Abstract

Emotions play an important role in shaping aggressive behavior, and understanding their underlying psychological mechanisms is particularly relevant among college students. However, existing research has predominantly focused on reactive aggression, while comparatively less attention has been paid to proactive aggression, which is more instrumental in nature and associated with more severe social consequences. In addition, empirical evidence regarding the valence-specific effects of awe remains limited. The present study aimed to examine the differential effects of positive and negative awe on proactive aggression and to explore the role of empathy as a potential mediating mechanism. A total of 110 college students were randomly assigned to one of three conditions: positive awe, negative awe, or neutral emotion. Awe was induced through video clips depicting natural landscapes. Proactive aggression was assessed using a modified bug-killing paradigm, including two behavioral indicators: force intensity and proportion of bugs killed. Empathy was measured using the Interpersonal Reactivity Index. The results revealed a clear differentiation based on the valence of awe. Participants in the positive awe condition exhibited significantly lower levels of proactive aggression than those in the neutral condition across both force intensity (M = 2.86, SD = 0.81 vs. M = 4.17, SD = 0.81) and proportion of bugs killed (M = 0.68, SD = 0.25 vs. M = 0.93, SD = 0.11). In contrast, the inhibitory effects of negative awe were weaker and less consistent. Compared with the neutral condition, negative awe was associated with a lower proportion of bugs killed, although this effect only reached marginal significance (*p* = 0.06, η^2^ = 0.04), and no significant difference was observed for force intensity. Mediation analyses indicated that empathy partially mediated the association between positive awe and proactive aggression. Empathy accounted for 31% of the total effect in the force intensity pathway (*B* = −0.02, *t* = −4.25, *p* < 0.001, 95% CI [−0.04, −0.01]) and 18% in the proportion-of-bugs-killed pathway (*B* = −0.003, *t* = −2.37, *p* = 0.02, 95% CI [−0.006, −0.001]). Notably, no significant mediating effect of empathy was observed in the negative awe condition, suggesting that the psychological processes linking awe to proactive aggression may differ as a function of emotional valence. Taken together, the present findings suggest that positive awe is reliably associated with lower levels of proactive aggression among college students, and that this association is partially explained by increased empathy. By contrast, the effects of negative awe appear to be fragile and context-dependent, as reflected in their failure to reach statistical significance, indicator-specific manifestation, and the absence of a consistent mediating pathway. These results highlight the importance of distinguishing between positive and negative awe when examining the behavioral consequences of self-transcendent emotions and underscore the need for further research to clarify the conditions under which negative awe may influence aggressive behavior.

## 1. Introduction

Aggression refers to the tendency or behavior of intentionally inflicting psychological or physical harm on others (Anderson & Bushman, 2002). A substantial body of research has demonstrated that aggressive behavior is associated with a range of adverse consequences, including psychological problems among victims (e.g., low self-esteem and depression), increased legal risks for perpetrators, and heightened tendencies toward antisocial behavior (Allen et al., 2018; Holekamp & Strauss, 2016). Based on differences in motivational origins, aggression is commonly classified into reactive aggression and proactive aggression (Miller & Lynam, 2006). Reactive aggression is characterized by impulsive, emotionally driven responses, often triggered by anger or frustration, whereas proactive aggression is instrumental in nature and involves deliberate, goal-oriented behaviors enacted to achieve specific outcomes (Merk et al., 2005). Accumulating evidence suggests that these two forms of aggression are underpinned by partially distinct psychological mechanisms. Specifically, reactive aggression has been shown to be closely associated with impulsivity, emotional dysregulation, and deficits in executive functioning (Polman et al., 2007), whereas proactive aggression is more strongly linked to chronic experiences of frustration and relative deprivation (Hubbard et al., 2010). These findings indicate that reactive and proactive aggression should not be treated as functionally equivalent constructs.

Despite this distinction, existing research has disproportionately focused on reactive aggression, with proactive aggression receiving comparatively limited empirical attention. Correspondingly, many commonly used experimental paradigms—such as the Competitive Reaction Time Task and electric shock punishment tasks—are primarily designed to capture affect-driven, immediate aggressive responses. As a result, these paradigms may be insufficient for assessing instrumental and premeditated forms of aggression that characterize proactive aggression (Ritter & Eslea, 2005; Tedeschi & Quigley, 1996). Importantly, proactive aggression is often more covert and persistent in nature, which may render its long-term consequences for individuals and society particularly severe. To address this limitation, the present study adopts a virtual-context paradigm to simulate proactive aggression, employing a modified bug-killing task with two behavioral indicators—force intensity and proportion of bugs killed—to more comprehensively assess instrumental aggressive behavior (Martens et al., 2007). By doing so, this study aims to explore potential psychological factors that may buffer proactive aggression.

Previous research on aggressive behavior has primarily focused on children and adolescents, whereas relatively few studies have examined this issue among college students (Buchmann et al., 2013; Lieberman et al., 1999). College students are in a critical stage of psychosocial development, during which the antecedents and manifestations of aggressive behavior may be distinct from those observed in younger populations (Thomas, 2019). This developmental period is also pivotal for the formation of a healthy personality. Within the university context, students are frequently exposed to interpersonal conflicts, academic and competitive pressures, and online aggression, all of which may serve as situational triggers for aggressive behavior. At the same time, college students exhibit considerable psychological plasticity, suggesting that their behavioral tendencies can be effectively shaped through interventions such as positive emotion induction. Therefore, investigating aggressive behavior among college students not only contributes to a deeper understanding of its underlying psychological mechanisms but also provides important implications for mental health education in higher education settings (Nocera & Dahlen, 2020).

Current psychological research on aggressive behavior has primarily examined demographic, cognitive, and emotional factors. A review of the evolution of aggression theories suggests that emotional factors are playing an increasingly prominent role in understanding aggressive behavior. Early biological theories (Berkowitz, 2012) posited that aggression originates from innate drives rather than learning processes; however, these perspectives cannot adequately explain the substantial variability of aggression across individuals and cultures. Frustration–aggression theory has undergone notable development over time (Kruglanski et al., 2023). Dollard et al. (1939) initially proposed a single-factor model, arguing that aggression is always a consequence of frustration. In contrast, Berkowitz (1989) later suggested that frustration is not the sole antecedent of aggression and that additional factors mediate the transition from frustration to aggressive responses. With the advancement of cognitive psychology, subsequent models incorporated emotion–cognition interaction mechanisms, highlighting the growing recognition of emotional processes in shaping aggressive behavior. Social learning theory (Nabavi & Bijandi, 2012) emphasizes the roles of direct reinforcement and observational learning in the acquisition of aggression. Sociocultural contexts also can indirectly influence individual behavior through the interaction between emotional arousal and cognitive appraisal (Khadka, 2024). Taken together, the progression from biological to social-cognitive theories indicates that emotion has been increasingly established as a central regulatory factor in aggression research. Consistent with this theoretical trend, empirical studies have identified emotion as a key predictor of aggressive behavior (Liu et al., 2022). For example, depressive symptoms have been found to be positively associated with aggression (Dutton & Karakanta, 2013), and individuals experiencing anger are more likely to engage in aggressive acts (Lochman et al., 2024).

In recent years, the self-transcendent positive emotion of awe has attracted growing attention in the study of social behavior (Keltner & Haidt, 2003). Commonly defined as a response to vast stimuli that challenge existing mental frameworks, awe requires individuals to undergo cognitive accommodation to integrate the experience. Experiencing awe tends to induce a “small self,” reducing self-centeredness and dominance motives while enhancing feelings of connectedness to others and the broader social world (Piff et al., 2015). These psychological shifts are theoretically relevant to aggression because aggressive behavior is often fueled by ego-threat, entitlement, and heightened anger. Empirical evidence suggests that awe can attenuate hostile tendencies and aggressive responding, particularly under provocation, by dampening anger and increasing self-control and prosocial orientation (Jiang et al., 2024). Moreover, awe has been shown to foster humility, empathy, and moral concern—processes that are inversely related to interpersonal aggression (Prade & Saroglou, 2016). Taken together, these findings imply that awe may function as a protective emotional state that buffers individuals against aggressive impulses, yet its role in aggression—especially in emerging adults and digital contexts—remains underexplored.

Awe is not a unitary emotion; rather, it can be differentiated into positive awe and negative awe based on its valence (Gordon et al., 2017; Stellar & Gordon, 2024). As Keltner and Haidt (2003) noted, awe occupies a boundary region between positive and negative affective states. Although awe is predominantly characterized by positive feelings, it also contains elements of fear, anxiety, and threat (Piff et al., 2015; van Elk et al., 2016). This dual nature can be seen in everyday life. Positive awe is often elicited by majestic natural landscapes or inspiring moral exemplars, whereas negative awe may arise from overwhelming natural forces or destructive abuses of power. While much of the positive psychology literature has emphasized the beneficial aspects of awe (Chen & Mongrain, 2021), recent studies indicate that awe may also evoke anxiety and defensive responses (Chaudhury et al., 2021). This duality raises an important theoretical question about whether awe inhibits or facilitates aggressive behavior. Addressing this issue requires distinguishing the differential effects of positive versus negative awe (Gordon et al., 2017; Stellar & Gordon, 2024).

Positive awe is thought to reduce self-centered cognition and enhance the “prosocial small self,” thereby attenuating aggressive motivation (Yang et al., 2016). In contrast, although negative awe may temporarily suppress overt aggression, it may also generate sustained anxiety that could accumulate over time and manifest as covert or indirect aggression (Anderson & Bushman, 2002). Prior research has generally shown that both positive and negative awe can diminish self-focus and foster a sense of small self, which tends to increase tolerance and promote prosociality while reducing aggression (Prade & Saroglou, 2016). To strengthen theoretical coherence, we clarify that the ‘small self’ operates as a valence-general cognitive process. However, the behavioral consequences of this process may be valence-specific. Nevertheless, the inhibitory effect of awe on aggression appears to differ across valences. In particular, positive awe has been shown to exert a stronger buffering effect than negative awe (Gordon et al., 2017).

Based on this evidence, the present study proposes:

**Hypothesis** **1.** *Positive awe and negative awe exert differential inhibitory effects on proactive aggression among college students. Compared with a neutral emotional state, both forms of awe are expected to reduce proactive aggressive tendencies, but the inhibitory effect of positive awe will be significantly stronger than that of negative awe.*

Regarding the pathways through which awe influences aggressive behavior, most existing studies have primarily focused on the mediating role of the “threatened small self.” These studies generally suggest that, regardless of whether awe is experienced as positively or negatively valenced, it tends to reduce aggression by eliciting a diminished sense of self. However, it remains unclear whether additional mediating mechanisms are involved. While self-control and fear may also function as mediators, empathy provides a more theoretically consistent link to the self-transcendent nature of awe. Self-control typically involves effortful regulation that suppresses impulses, whereas empathy reflects a shift in attention away from the self and toward the experiences of others, consistent with the “small self” associated with awe (Piff et al., 2015). In contrast, fear remains primarily self-focused and may motivate avoidance rather than concern for others. Empathy, however, promotes prosocial motivation that can support sustained reductions in aggression. In the case of proactive aggression, empathy targets the underlying lack of concern for others rather than simply inhibiting aggressive responses. Accordingly, empathy offers a plausible mechanism through which reduced self-focus translates into greater prosocial orientation. Yang et al. (2016) found that positive awe can inhibit aggressive tendencies by enhancing empathy, yet it is still uncertain whether a similar mechanism operates under negative awe (Gao et al., 2025). Empathy, defined as the capacity to understand others’ experiences through affective resonance (Telle & Pfister, 2016), has been consistently identified as a key driver of prosocial behavior, including volunteering and helping actions (Bartal et al., 2011). Fredrickson’s (2001) broaden-and-build theory further suggests that self-transcendent positive emotions such as awe can replenish psychological resources and alleviate empathic depletion. This theoretical account aligns with empirical findings showing that awe positively predicts empathic capacity (Morelli et al., 2015), as well as neuroimaging evidence indicating functional coupling between neural networks associated with awe (e.g., default mode network suppression and frontoparietal activation) and those involved in empathy (Marsh, 2018). Accordingly, the present study systematically examines empathy as a potential mediator in the relationship between awe and aggression and proposes:

**Hypothesis** **2.** *Positive awe reduces proactive aggression by enhancing empathy, whereas this mediating pathway is expected to be weaker or absent for negative awe.*

## 2. Methods

### 2.1. Participants

An a priori power analysis was conducted using G*Power 3.1 (Faul et al., 2007; Heinrich Heine University Düsseldorf, Düsseldorf, Germany) for a one-way ANOVA (fixed effects, omnibus, one-way). Based on an assumed medium-to-large effect size (*f* = 0.30), an alpha level of 0.05, and a power of 0.80, the analysis indicated that a total of *N* = 111 participants was necessary. Accordingly, 110 undergraduate students were recruited from a university in Shandong Province, China. Participants were randomly assigned to one of three experimental conditions, including positive awe, negative awe, and neutral emotion. After excluding five participants due to incomplete responses or careless responding, the final sample consisted of 105 valid participants (35 in each condition), which provided an achieved power of approximately 0.77, indicating acceptable statistical sensitivity. The sample included 54 males and 51 females. All participants reported no prior involvement in similar studies, were right-handed, and had normal or corrected-to-normal vision. All experiments were approved by the Human Research Ethics Committee of University of Jinan (UJN2024-022), and written informed consents were obtained from all subjects. All participants received a gift worth 5 Chinese yuan after the experiment.

Preliminary analyses indicated no significant gender differences in the primary dependent variables. Given that the study was not specifically powered to detect interaction effects between gender and experimental conditions (G*Power indicated that such an interaction would require a significantly larger n than the current sample), gender was not included as a moderator in the final mediation models.

### 2.2. Experimental Design

The experiment employed a single-factor between-subjects design. The independent variable was emotion type, which consisted of three levels: namely, positive awe, negative awe, and neutral emotion. Participants were randomly assigned to one of the three conditions with equal allocation.

The primary dependent variable was aggressive behavior, operationalized through performance on a simulated insect-killing task. Consistent with prior research (Buckels et al., 2013; Giancola & Parrott, 2008), the assessment involved two behavioral indices, including the proportion of insects killed, which was defined as the percentage of insects selected for elimination out of the total number presented; and mean level of force selected, reflecting the average intensity of force participants chose when “killing” the insects. Higher values on both indices indicate greater aggressive tendency. To control for baseline individual differences in aggression, pretest scores of both aggression indices were entered as covariates in subsequent analyses and statistically controlled using analysis of covariance (ANCOVA).

### 2.3. Materials

#### 2.3.1. Emotion Induction and Manipulation Check

##### Emotion Induction Materials

Following Piff et al. (2015), a video-based induction paradigm was used to elicit target emotions through visual stimuli. Participants in the positive awe condition viewed a composite clip produced by the British Broadcasting Corporation (BBC) depicting vast plains, forests, and aurora borealis. Participants in the negative awe condition watched a BBC composite clip featuring powerful natural disasters, including volcanoes, tsunamis, and tornadoes. Prior empirical studies have demonstrated that these materials effectively elicit awe-related emotions among Chinese young adults (Yang et al., 2016). Participants in the neutral control condition viewed a standardized eye-exercise instructional video, which has been validated as emotionally neutral in previous research (Samide et al., 2020). All videos were presented under strictly standardized conditions, and the duration of each clip was uniformly set to 2 min.

##### Emotion Measurement and Manipulation Check

Participants’ emotional responses were assessed using a 7-point Likert scale adapted from the emotional state self-report measure developed by Gross and Levenson (1995). Immediately after viewing the video, participants rated the extent to which they experienced six core emotions: awe, wonder, happiness, joy, fear, and anxiety (1 = *not at all*, 7 = *very much*). Consistent with Chaudhury et al. (2021), composite indices were created as follows: the awe score was calculated as the mean of the awe and wonder items (Cronbach’s α = 0.88); the positive emotion score was computed as the mean of the happiness and joy items (Cronbach’s α = 0.91); and the negative emotion score was derived from the mean of the fear and anxiety items (Cronbach’s α = 0.89). The manipulation was considered successful when three criteria were met. Participants in both awe conditions (positive and negative) reported significantly higher awe scores than those in the neutral condition; participants in the negative awe condition showed significantly higher negative emotion scores than the other two groups; and participants in the positive awe condition exhibited significantly higher positive emotion scores than the other two groups. These criteria jointly indicate both the effectiveness and specificity of the emotion induction.

##### Proactive Aggression

Proactive aggression was assessed using a modified insect-killing paradigm originally developed by Martens et al. (2007). Building on this paradigm, we replaced real insect-killing with a computerized simulation in which participants selected both the number of insects to be killed and the level of force used on a computer screen. In the task, participants were told that they would complete a simple simulation of pest control. On each trial, an image depicting a certain number of insects appeared on the screen, and the total number of insects was clearly labeled. Participants were first asked to select a level of force from *1* (very mild) to *5* (very strong) using the mouse. They then entered the number of insects they wished to eliminate, which could not exceed the number displayed on the screen. After reading standardized instructions, participants completed five practice trials to familiarize themselves with the procedure, followed by 40 formal experimental trials. To control for baseline individual differences in proactive aggression, a pretest–posttest design was adopted wherein participants performed the insect-killing task once before emotion induction and once again after the induction. Two behavioral indices were derived as dependent variables. First, mean force level across trials was used as an indicator of aggression intensity (range = 1–5). Second, the proportion of insects killed was calculated as the total number of insects selected for elimination divided by the total number presented across all trials (420 insects in total; range = 0–1). Higher values on both indices indicated greater proactive aggressive tendency.

##### Empathy

Empathy was assessed using the Chinese version of the Interpersonal Reactivity Index (IRI) (Y. Zhang, 2025). The scale consists of 22 items across four subscales: Perspective-Taking (assessing the cognitive ability to understand others’ viewpoints), Empathic Concern (capturing feelings of warmth and compassion for others in distress), Fantasy (reflecting the tendency to imaginatively transpose oneself into fictional situations), and Personal Distress (measuring anxiety and discomfort in tense interpersonal contexts). Participants rated each item on a 5-point Likert scale ranging from 0 (*does not describe me well*) to 4 (*describes me very well*). Higher total scores indicate greater overall empathic capacity. Items 2, 5, 10, 11, and 14 were reverse-scored prior to computing the total score. The Chinese version of the IRI has demonstrated good internal consistency, with a reported Cronbach’s α of 0.82 (F. F. Zhang et al., 2010).

##### Procedures

The experiment was programmed and presented using PsychoPy (version 2024.2.5; Peirce et al., 2019; University of Nottingham, Nottingham, UK), a widely used open-source platform for psychological experimentation. The overall procedure consisted of four sequential phases. In Phase 1, participants read standardized instructions to understand the task requirements. They then completed five practice trials of the insect-killing task, followed by the formal pretest of proactive aggression, which comprised 40 trials. In Phase 2, participants wore headphones and attentively watched the assigned emotion-induction video corresponding to their experimental condition. Immediately afterward, they completed six manipulation-check items assessing their emotional responses. In Phase 3, the instructions for the insect-killing task were presented again. After familiarization, participants completed another five practice trials, followed by the posttest of proactive aggression consisting of 40 trials. In Phase 4, participants completed the Chinese version of the Interpersonal Reactivity Index (IRI), which includes 22 items, five of which are reverse-scored. In each insect-killing trial, participants performed two sequential operations. First, they selected a force level ranging from 1 to 5. Second, they entered the number of insects to be eliminated, with the number of insects on the screen randomly varying between 1 and 20 per trial; participants could not select a number exceeding the amount currently displayed. Across the 40 formal trials, the total number of insects presented was 420.

##### Data Analysis Strategy

All statistical analyses were conducted using IBM SPSS Statistics 26.0 (IBM Corp., Armonk, NY, USA). The analytic procedure consisted of three steps. First, one-way between-subjects ANOVA followed by post hoc multiple comparisons was used to examine whether the emotion induction was successful and whether significant differences existed among the three emotion conditions. Second, two separate analyses of covariance (ANCOVAs) were performed to test the effects of emotion type on the two aggression indicators, with their respective pretest scores entered as covariates. Effect sizes were reported to evaluate the magnitude of group differences. Third, the mediating role of empathy was tested using the PROCESS macro for SPSS (Model 4; Hayes, 2018), employing a bias-corrected bootstrap procedure with 5000 resamples and 95% confidence intervals. This mediation analysis was conducted separately for each dependent variable, while simultaneously controlling for the corresponding pretest covariate, in order to examine whether empathy significantly mediated the relationship between emotion type and proactive aggression in both outcome pathways.

## 3. Results

### 3.1. Manipulation Check for Emotion Induction

To examine the effectiveness of the emotion induction, one-way between-subjects ANOVAs followed by post hoc multiple comparisons were conducted (see Table 1). Results indicated significant differences among the groups on the awe, positive emotion, and negative emotion dimensions. On the awe dimension, the positive awe group (M = 10.51, SD = 2.61) and the negative awe group (M = 9.97, SD = 2.03) did not differ significantly, *p* = 0.28; however, both groups scored significantly higher than the neutral group (M = 4.46, SD = 1.52), *p* < 0.001. On the positive emotion dimension, the positive awe group (M = 9.80, SD = 3.00) scored significantly higher than the negative awe group (M = 4.29, SD = 1.76) and the neutral group (M = 5.94, SD = 2.40), *p* < 0.001. The neutral group showed marginally higher positive emotion than the negative awe group, *p* = 0.05. On the negative emotion dimension, the negative awe group (M = 9.11, SD = 2.69) scored significantly higher than the positive awe group (M = 3.74, SD = 1.88) and the neutral group (M = 3.94, SD = 1.47), *p* < 0.001. No significant difference was found between the positive awe and neutral groups on negative emotion, *p* = 0.68.

Taken together, these results indicate that the emotion induction successfully elicited distinct and specific emotional states corresponding to positive awe, negative awe, and neutral conditions.

### 3.2. Effects of Emotion on Proactive Aggression

Given the significant correlation between the two dependent variables (r = 0.54, *p* < 0.001), a multivariate analysis of covariance (MANCOVA) was initially performed to strictly control the type I error rate. The multivariate results confirmed a significant overall effect of the experimental conditions, Wilks’ Lambda = 0.637, *F* (4, 198) =12.535, *p* < 0.001. Following this significant omnibus test, separate univariate ANCOVAs were conducted for the proportion of insects eliminated and the mean force level to further elucidate the specific impact of emotion induction on distinct dimensions of aggressive behavior.

Although Levene’s test indicated unequal variances across conditions (*p*s < 0.01), we maintained the ANCOVA framework because the F-statistic is remarkably robust to heteroscedasticity when group sizes are perfectly balanced (Box, 1954; Harwell, 1992; Schmider et al., 2010). With an equal sample size of *n* = 35 per condition, the risk of inflating Type I error due to variance inequality is considered negligible in such a balanced design. Regarding the homogeneity of regression slopes, the interaction between the emotion condition and pretest covariates was non-significant for both mean force level (*F* (2, 99) = 1.631, *p* = 0.201) and proportion of bugs killed (*F* (2, 99) = 0.976, *p* = 0.381), confirming that the relationship between pretest and posttest scores remained consistent across groups. To examine the effects of emotion on proactive aggression while controlling for baseline aggression, one-way ANCOVAs were conducted with the respective pretest scores as covariates. Separate analyses were performed for the two behavioral indicators, mean force level and proportion of insects eliminated (see Table 2).

When baseline mean force level was entered as a covariate and post-test mean force level served as the dependent variable, post hoc pairwise comparisons revealed that participants in the positive awe condition (M = 2.86, SD = 0.81) exhibited significantly lower levels of proactive aggression than those in both the neutral condition (M = 4.17, SD = 0.81) and the negative awe condition (M = 3.80, SD = 0.83), *p* < 0.001, η^2^ = 0.23. In contrast, the difference between the neutral and negative awe conditions was not significant, *p* = 0.21, η^2^ = 0.02.

When baseline proportion of insects eliminated was included as a covariate and post-test proportion of insects eliminated was the dependent variable, post hoc tests again indicated that proactive aggression was significantly lower in the positive awe condition (M = 0.68, SD = 0.25) than in both the neutral condition (M = 0.93, SD = 0.11) and the negative awe condition (M = 0.86, SD = 0.17), *p* < 0.001, η^2^ = 0.31. Moreover, participants in the negative awe condition tended to eliminate fewer insects than those in the neutral condition, a difference that did not reach statistical significance, *p* = 0.06, η^2^ = 0.04.

Taken together, these findings demonstrate a cross-indicator consistency in the effect of emotional states on proactive aggression. Specifically, positive awe reliably reduced proactive aggression across both behavioral indices, whereas the inhibitory effect of negative awe varied depending on the measurement indicator.

### 3.3. Mediation Analysis: The Role of Empathy

To examine whether empathy mediated the effect of emotional condition on proactive aggression, we conducted mediation analyses using PROCESS (Model 4) within a covariate-adjusted framework. Because the independent variable (emotional condition) was a three-level nominal variable (positive awe, negative awe, neutral), two dummy variables were created in SPSS 26.0: D1 compared the positive awe condition (coded 1) with the neutral condition (coded 0), and D2 compared the negative awe condition (coded 1) with the neutral condition (coded 0). Baseline levels of the corresponding aggression indicators were included as covariates in all models.

**Figure 1:**
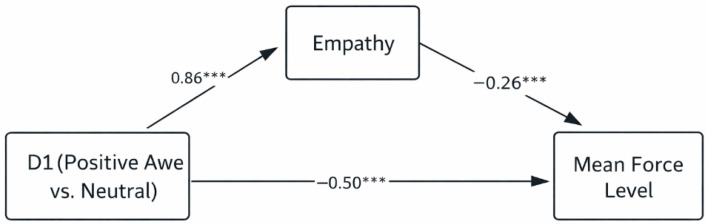
Mediation model of empathy in the relationship between positive awe and mean force level. *** *p* < 0.001.

**Figure 2:**
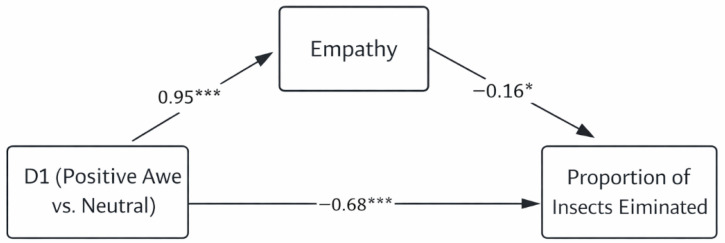
Mediation model of empathy in the relationship between positive awe and proportion of insects eliminated. * *p* < 0.05; *** *p* < 0.001.

Results for the mediation of positive awe (D1) are illustrated in Figure 1 and Figure 2, and summarized in Table 3 and Table 4. D1 significantly and positively predicted empathy (force-level model: *B* = 8.88, *t* = 4.50, *p* < 0.001; proportion-of-insects-eliminated model: *B* = 9.80, *t* = 5.15, *p* < 0.001). In turn, empathy significantly and negatively predicted both indicators of proactive aggression (force level: *B* = −0.02, *t* = −4.25, *p* < 0.001; proportion eliminated: *B* = −0.003, *t* = −2.37, *p* = 0.02).

Bootstrap analyses (5000 resamples, 95% confidence intervals) indicated that D1 had a significant total effect on aggression (force level: *B* = −0.71, *t* = −5.62, 95% CI [−0.95, −0.46]; proportion eliminated: *B* = −0.17, *t* = −6.53, 95% CI [−0.23, −0.12]). The direct effect of D1 remained significant after accounting for empathy (force level: *B* = −0.49, *t* = −3.82, 95% CI [−0.74, −0.23]; proportion eliminated: *B* = −0.14, *t* = −4.87, 95% CI [−0.20, −0.08]). Critically, the indirect effect via empathy was significant for both aggression indicators (force level: *B* = −0.02, 95% CI [−0.04, −0.01]; proportion eliminated: *B* = −0.003, 95% CI [−0.006, −0.001]). The mediated pathway accounted for approximately 31% of the total effect in the force-level model and 18% in the proportion-eliminated model.

Overall, relative to the neutral condition, exposure to positive awe reduced proactive aggression in both behavioral indices through a significant partial mediation by empathy.

In contrast, the indirect effects of negative awe (D2) through empathy were not statistically significant for either aggression indicator (force level: 95% CI = [−0.21, 0.05]; proportion of insects eliminated: 95% CI = [−0.04, 0.01]), indicating that empathy did not significantly mediate the relationship between negative awe and proactive aggression.

Taken together, these findings suggest that the buffering effect of positive awe on proactive aggression is robust and consistent across behavioral indicators via an empathic pathway, whereas the potential influence of negative awe does not appear to operate through empathy. This pattern implies that any attenuating effect of negative awe on aggression may rely on alternative psychological mechanisms not assessed in the present study (e.g., threat sensitivity, fear-based inhibition, or self-protective withdrawal), or that the effect may be too small to be detected given current statistical power.

## 4. Discussion

This study systematically examined the differential effects of positive and negative awe on proactive aggression, including the mediating mechanism of empathy and its inhibitory effect on aggression across different valences. Using a video-based objective manipulation to induce awe of varying valences and a simulated bug-killing paradigm to measure proactive aggression, we investigated the impact of awe states on college students’ aggressive behavior and the mediating role of empathy. The results indicated that while both positive and negative awe inhibited proactive aggression to some extent, the inhibitory effect of positive awe was stronger than that of negative awe. Specifically, positive awe significantly reduced aggression levels across both dependent variable indicators. In contrast, the inhibitory effect of negative awe lacked statistical significance regarding the proportion of bugs killed. Furthermore, empathy partially mediated the relationship between positive awe and aggression across both indicators; however, this mediation pathway was not significant for negative awe, a finding that aligns with previous research on valence-specific emotional effects (e.g., Yang et al., 2016).

### 4.1. Theoretical Implications

Theoretically, these findings support the application of the emotional valence differentiation theory in awe research. While early research often conceptualized awe as a unidimensional positive emotion (Keltner & Haidt, 2003), recent studies have increasingly emphasized its valence-differentiated characteristics (Gordon et al., 2017). In the present study, positive awe induced by natural landscapes significantly inhibited aggressive behavior (Yang et al., 2016), whereas negative awe showed no inhibitory effect on a specific indicator. This conclusion aligns with the core tenets of the valence differentiation perspective (Stellar & Gordon, 2024). A key theoretical contribution of this study is clarifying why these valences diverge through the lens of the ‘small self’. While both positive and negative awe involve a reduced sense of self, they appear to produce qualitatively different self-states. Positive awe, often elicited by natural beauty or vastness, fosters a prosocial small self marked by feelings of connectedness and oneness, which provides an affective basis for empathic concern. In contrast, negative awe, typically associated with threat-based appraisals (Gordon et al., 2017), evokes a threatened small self accompanied by anxiety and personal distress, responses that are self-focused and distinct from other-oriented empathy. As a result, whereas positive awe may facilitate empathic engagement, negative awe may instead be linked to defensive self-preservation, potentially reducing empathic responses that inhibit proactive aggression. Such a pattern is consistent with the absence of a significant mediation pathway for negative awe observed in the present study.

The pathway for positive awe appears to operate through the enhancement of empathy, whereby elevated empathy leads to affective resonance, which subsequently reduces aggressive motivation (Telle & Pfister, 2016). Conversely, while negative awe also triggers the experience of awe, the accompanying negative affect may interfere with the effective allocation of cognitive resources (van Elk et al., 2016). This distinction further elucidates why empathy specifically buffers against the instrumental nature of proactive aggression (PA). Although PA is theoretically ‘cold’ and instrumental (Raine et al., 2006), it remains susceptible to emotional modulation. We contend that the ‘calculated’ nature of PA stems from an affective valuation deficit, where the victim’s welfare is excluded from the individual’s subjective cost–benefit analysis. Awe addresses this by inducing a ‘small self’ that restores social attunement and re-introduces the ‘affective cost’ of harm (Martens et al., 2007). Unlike the top-down inhibition of self-control, awe-induced empathy facilitates a bottom-up motivational shift that recalibrates the decision-making calculus, rendering aggression less ‘profitable.’ This mechanism explains the superior inhibitory effect of positive awe, in that while both awe valences elicit a valence-general cognitive shift in self-perception, positive awe more effectively replenishes the empathic resources required to counteract the instrumental drive behind proactive aggression.

### 4.2. The Paradox of Negative Awe

Focusing specifically on negative awe, this study reveals its complex and potentially contradictory nature. Empirically, our results showed that while negative awe trended toward reducing aggression, the inhibitory effect on the ‘proportion of bugs killed’ was non-significant, and the mediation path via empathy remained absent. These findings lead us to propose a ‘double-edged sword’ conjecture regarding the impact of negative awe. On one hand, we hypothesize that the deterrent power of negative awe (e.g., disaster scenes) may briefly inhibit aggression through risk aversion. Drawing on the survival instinct, perceived threats may prioritize group cooperation over aggressive impulses (Smith et al., 2014), a process potentially driven by a threatened small self that focuses on immediate self-preservation. On the other hand, we offer a post hoc interpretation that the anxiety inherent in negative awe may simultaneously deplete cognitive resources. This cognitive depletion, a mechanism suggested but not directly measured in our study (van Elk et al., 2016), could impair an individual’s capacity for emotion regulation. This may explain the observed discrepancy where participants reduced the number of bugs killed (a simpler decision) but failed to demonstrate equivalent restraint in force level (a more impulsive physical response). Furthermore, we tentatively suggest a temporal decay mechanism to explain these results, suggesting that while the initial deterrence of negative awe might produce a short-term inhibitory effect, prolonged exposure or the high arousal of a threatened small self may lead to emotional exhaustion. This could cause the inhibitory effect to decay or even reverse over time. Future research utilizing direct measures of cognitive load and physiological arousal is required to empirically test these theoretical conjectures.

### 4.3. Methodological Contributions

From a methodological perspective, the experimental paradigm employed in this study offers innovative significance (Martens & Kosloff, 2012; Tedeschi & Quigley, 1996). Most prior research has relied on traditional paradigms measuring reactive aggression, such as the Competitive Reaction Time Task (Elson et al., 2014) or the aggression paradigm involving electric shocks (Zeichner et al., 1999). However, these traditional paradigms do not adequately reflect proactive aggression levels. Consequently, this experiment adapted the bug-killing paradigm by Martens et al. (2007) using PsychoPy 2024.2.5 software. By programming multiple trials involving decisions on force levels and the number of bugs to exterminate, we successfully simulated a scenario of proactive aggression (Martens et al., 2007). The results showed high effect sizes for the positive awe group across both aggression indicators, and the mediation paths were consistent across indicators. This validates the effectiveness of the modified simulated bug-killing paradigm in measuring proactive aggression. Additionally, by using pre-test aggression scores as covariates to control for baseline levels, the experiment effectively isolated the specific effects of the emotional induction manipulations.

### 4.4. Practical Implications

This study also holds significant practical implications, particularly regarding new approaches for the prevention and intervention of violence among college students (Coker & Clear, 2014; Kovalenko et al., 2022). The results suggest that positive awe can serve as an effective psychological intervention resource. Therefore, it is valuable for higher education mental health programs to design nature-experience curricula to cultivate a sense of positive awe, such as organizing outdoor activities or utilizing technology to create virtual reality scenarios of grand landscapes (Chirico et al., 2018). Regarding negative awe, educators must be vigilant about its potential negative impacts (Jiang et al., 2024). Although disaster documentaries may briefly inhibit aggression, the anxiety they provoke cannot be ignored. This anxiety may act as a catalyst for increased psychological stress; long-term exposure to negative awe could lead to emotional exhaustion or moral numbness (Gordon et al., 2017). Consequently, in mental health education activities, educators should prioritize materials that induce positive awe. If materials inducing negative awe are unavoidable, educators must fully assess the potential negative impact and design post hoc cognitive reappraisal training to mitigate these effects (Gordon et al., 2017).

### 4.5. Limitations and Future Directions

Several limitations of this study should be addressed in future research. First, sample homogeneity may limit the generalizability of the findings. The participants were all from the same university in Shandong, sharing relatively uniform values and developmental environments. However, the experience and expression of awe can differ significantly across cultures; for instance, collectivist cultures tend to emphasize the social norm functions of awe (Yang et al., 2016). Future research should expand the sampling range to compare responses among college students from different regions or cultural backgrounds.

Second, the ecological validity of the aggression measurement requires improvement. Although the simulated bug-killing paradigm is more effective than traditional paradigms for measuring proactive aggression, a gap remains between the laboratory setting and real-world aggressive situations (Giancola & Parrott, 2008; Tedeschi & Quigley, 1996). The behavioral task used in the present study should therefore be interpreted as an indirect indicator of aggressive tendencies rather than a direct measure of interpersonal aggression. Future studies could consider mixed experimental designs, such as employing post-test tracking of daily behaviors, to enhance external validity (Leviton, 2017).

Finally, this study found that the mediation path of empathy was only supported (as partial mediation) in the positive awe condition; the mechanism for negative awe remains unclear. It should be noted that the calculated mediation percentages (e.g., 31% and 18%) may exhibit instability due to our sample size. These values should be interpreted as descriptive indicators rather than precise population parameters; the robustness of our model is primarily supported by the significant bootstrapped indirect effects. This null result may be due to small effect sizes requiring a larger sample power, or it may be that empathy is not the mediator for negative awe. Future research should increase sample sizes and consider introducing other psychological or physiological indicators (e.g., anxiety, cognitive load) to uncover potential pathways by which negative awe influences aggression (Yang et al., 2016). In summary, future scholarship should consider the simultaneous influence of multiple variables, introduce relevant moderators, diversify samples, enhance ecological validity, and utilize modern neuroscientific techniques to explore the underlying neurophysiological mechanisms.

## Figures and Tables

**Table 1:** Descriptive Statistics and ANOVA Results for Emotional Experience Across Conditions (M ± SD).

Dependent Variable	Positive Awe	Negative Awe	Neutral	F	η^2^
Awe	10.51 ± 2.61	9.97 ± 2.03	4.46 ± 1.52	89.03 ***	0.64
Positive Emotion	9.80 ± 3.00	4.29 ± 1.76	5.94 ± 2.40	47.11 ***	0.48
Negative Emotion	3.74 ± 1.88	9.11 ± 2.60	3.94 ± 1.47	77.32 ***	0.60

Note. Degrees of freedom for all ANOVAs: *df* = 102. *** *p* < 0.001.

**Table 2:** Descriptive statistics and ANCOVA results for proactive aggression across emotion conditions.

Outcome Variable	Positive Awe	Negative Awe	Neutral	*F* (2, 101)	η^2^
Mean force level	2.86 (0.81)	3.80 (0.83)	4.17 (0.81)	22.47 ***	0.25
Proportion of insects eliminated	0.68 (0.25)	0.86 (0.17)	0.93 (0.11)	23.69 ***	0.32

Note. Values are adjusted means (raw standard deviations in parentheses), *** *p* < 0.001.

**Table 3:** Mediation Analysis Results for the Mean Force Level.

Path	Effect	SE	95% CI	Proportion Mediated
Total effect	−0.71	0.13	[−0.95, −0.46]	-
Indirect effect	−0.22	0.08	[−0.39, −0.09]	31%
Direct effect	−0.49	0.13	[−0.74, −0.23]	69%

Note. Bootstrap sample size = 5000; bias-corrected 95% confidence intervals are reported.

**Table 4:** Mediation Analysis Results for proportion of insects eliminated.

Path	Effect	SE	95% CI	Proportion Mediated
Total effect	−0.17	0.03	[−0.23, −0.12]	-
Indirect effect	−0.03	0.01	[−0.06, −0.01]	18%
Direct effect	−0.14	0.03	[−0.20, −0.08]	82%

## Data Availability

The data presented in this study are available on request from the corresponding author.
